# Correlation between central venous oxygen saturation and mixed venous oxygen saturation in surgical patients: A systematic review and meta-analysis

**DOI:** 10.1016/j.aicoj.2026.100076

**Published:** 2026-05-12

**Authors:** Ling Li, Li-Xian He, Yun-Tai Yao, the Evidence in Cardiovascular Anesthesia (EICA) Group

**Affiliations:** a Department of Anesthesiology, Fuwai Yunnan Hospital, Chinese Academy of Medical Sciences, Affiliated Cardiovascular Hospital of Kunming Medical University, Kunming 650102, China; b Yunnan Provincial Cardiovascular Clinical Medical Center, Kunming 650102, China; c Yunnan Provincial Cardiovascular Clinical Medical Research Center, Kunming 650102, China; d Department of Anesthesiology, Fuwai Hospital, National Center for Cardiovascular Diseases, Peking Union Medical College and Chinese Academy of Medical Sciences, Beijing 100037, China; e Center for Outcomes Research, Department of Anesthesiology, Critical Care and Pain Medicine, University of Texas, Houston 77030, Texas, United States; f Outcomes Research Consortium, Houston 77030, Texas, United States

**Keywords:** Central venous oxygen saturation, Mixed venous oxygen saturation, Mean difference, Limits of agreement, Correlation coefficient

## Abstract

**Background:**

The validity of central venous oxygen saturation (ScvO_2_) as a surrogate for mixed venous oxygen saturation (SvO₂) in surgical patients remains uncertain.

**Methods:**

We systematically searched PubMed, Embase, the Cochrane Library, China National Knowledge Infrastructure (CNKI), Wanfang, VIP, and the Chinese BioMedical Literature & Retrieval System (SinoMed) up to March 31st 2026 for observational studies reporting paired perioperative ScvO_2_ and SvO_2_ measurements in surgical patients. Two reviewers independently screened studies, extracted data, and assessed quality using the Newcastle–Ottawa Scale (NOS). The primary outcome was clinical interchangeability, pre-defined as both the pooled mean difference (MD) 95% confidence interval (CI) and 95% limits of agreement (LOA) within ±5%. Correlation was a secondary outcome via Fisher's *z* transformation. For studies reporting multiple measurements within a single perioperative phase, arithmetic means were used to avoid unit-of-analysis errors. Random-effects models were prespecified for all pooled analyses, supplemented by pre-planned subgroup and sensitivity analyses to explore heterogeneity.

**Results:**

Twenty-eight studies including 1369 patients were analyzed. In studies reporting aggregated perioperative data (10 studies, *n* = 524), ScvO_2_ and SvO₂ were not clinically interchangeable: pooled MD was −0.49% (95% CI, −1.34 to 0.35; *I*² = 70.3%), and LOA (−12.15% to 11.17%) exceeded the predefined ±5% threshold for clinical acceptability, consistent with observations across perioperative phases. The pooled correlation was 0.72 (5 studies, *n* = 197; 95% CI 0.57–0.82; *I*² = 64.3%). Subgroup analyses showed narrower LOA and higher correlation in non-cardiovascular and off-pump procedures.

**Conclusions:**

ScvO_2_ is positively correlated with SvO₂ in the perioperative period. However, wide individual-level LOA indicate that the two measurements are not interchangeable in surgical patients. LOA are narrower in non-cardiovascular and off-pump procedures but remain outside clinically acceptable limits.

## Highlights

• ScvO_2_ is positively correlated with SvO₂ but shows limited reliability at the individual level.
• Wide LOA indicate ScvO_2_ cannot substitute for SvO₂ in surgical patients.
• Concordance is higher in non-cardiovascular and off-pump procedures, yet remains limited.

## Introduction

Mixed venous oxygen saturation (SvO₂), measured from pulmonary artery blood via pulmonary artery catheterization, has long been regarded as an important reference indicator for assessing the balance between systemic oxygen delivery and consumption in critically ill and surgical patients [1,2]. However, its measurement requires pulmonary artery catheterization, an invasive procedure associated with potential risks, including infection, arrhythmia, and pulmonary artery rupture [3].

Central venous oxygen saturation (ScvO_2_), obtained from a standard central venous catheter, has therefore emerged as a less invasive and more readily available alternative [4,5]. From a physiological perspective, SvO₂ reflects the integrated oxygen extraction from the entire body, whereas ScvO_2_, sampled predominantly from the superior vena cava, represents regional venous oxygenation mainly from the brain and upper extremities [1,6]. This anatomical and physiological distinction provides a mechanistic basis for potential dissociation between the two measurements.

Despite decades of investigation, clinical evidence regarding whether ScvO_2_ can reliably substitute for SvO_2_ remains inconsistent. While some studies have reported strong correlations between ScvO_2_ and SvO_2_ under specific conditions [[7], [8], [9]], others have demonstrated clinically relevant discrepancies, particularly during cardiopulmonary bypass (CPB) or in hemodynamically unstable states [[10], [11], [12]].

To address this uncertainty, we conducted a systematic review and meta-analysis to assess the clinical interchangeability of ScvO_2_ and SvO_2_ in surgical patients, providing quantitative evidence on the mean difference (MD) and 95% limits of agreement (LOA) between the two, based on predefined interchangeability criteria. Additionally, secondary analyses examined the correlation between ScvO_2_ and SvO_2_. Preplanned exploratory subgroup and sensitivity analyses were performed to identify potential sources of heterogeneity, including perioperative factors such as surgical type and the use of CPB.

## Methods

### Ethical approval

This meta-analysis was based exclusively on previously published studies; therefore, ethical approval and informed consent were not required, in accordance with the policy of the Ethical Committee of Fuwai Yunnan Hospital.

### Search strategy

This systematic review and meta-analysis was conducted in accordance with the Meta-analysis Of Observational Studies in Epidemiology (MOOSE) and the Preferred Reporting Items for Systematic Reviews and Meta-Analyses (PRISMA) guidelines [13,14], and the study protocol was prospectively registered in PROSPERO (CRD42022374046). Databases searched included PubMed, Cochrane Library, Ovid, Embase, CNKI, Wanfang, VIP, and SinoMed (from inception to October 31st, 2025, updated March 31st, 2026) using terms: “central venous oxygen saturation,” “ScvO_2_,” “mixed venous oxygen saturation,” “SvO_2_,” “SmvO_2_,” “relevance,” “correlation,” “agreement,” “relationship.” (Appendix A). No language or regional restrictions were applied. Reference lists were screened for additional studies.

### Inclusion and exclusion criteria

Studies were included if they met all of the following criteria: compared ScvO_2_ and SvO_2_ in surgical patients; reported MD and LOA; reported correlation coefficient (*r*); included ≥5 participants. Excluded were reviews, editorials, case reports, letters, conference abstracts, animal/in vitro studies, duplicates, non-surgical populations, or insufficient quantitative data. Two reviewers (LL and LXH) independently screened studies, with a third (YTY) resolving discrepancies.

### Data extraction and quality assessment

Two independent reviewers (LL and LXH) independently extracted data included study characteristics, patient demographics, perioperative context, monitoring methods, and reported outcome measures pairs, MDs, LOAs, and *r*. To ensure a comprehensive representation of the perioperative trajectory and mitigate selection bias, data from all reported clinical phases were extracted. Rather than selecting a single time point, for studies reporting multiple measurements within a single clinical phase, arithmetic means were calculated to derive a phase-level summary estimate. Additionally, information on the timing of paired sampling was systematically extracted to characterize measurement simultaneity. For non-English studies, translation tools were used solely to facilitate an understanding of the study context; all numerical data were verified directly from original-language tables and figures. A third reviewer (YTY) independently verified the extracted data, and discrepancies were resolved through consensus.

Study quality was assessed using the Newcastle–Ottawa Scale (NOS), with scores categorized as low (0–3), moderate (4–6), or high quality (7–9) [15].

### Statistical analysis

The statistical analysis evaluated two prespecified outcomes: clinical interchangeability, assessed using the pooled MD and 95% LOA (primary outcome), and the correlation coefficient (secondary outcome). Clinical interchangeability between ScvO_2_ and SvO_2_ was defined a priori as the condition in which both the 95% confidence interval (CI) of the pooled MD and the pooled 95% LOA lay entirely within a clinically acceptable margin of ±5% [11].

### Bland–Altman analysis

A two-stage meta-analytic approach was employed for the Bland-Altman analysis. First, study-specific MDs were pooled. Subsequently, within the Bland–Altman framework, the pooled 95% LOA were derived by combining the variability of the paired differences according to the following formula: pooled MD ± 1.96 × pooled *SDd*, where *SDd* denotes the standard deviation of the differences. *SDd* values were combined using a variance-weighted average, weighted by the corresponding degrees of freedom (*n*−1).

For the secondary outcome, correlation coefficients were synthesized using an inverse-variance-weighted random-effects meta-analysis of Fisher’s *z*-transformed values. The pooled *z* estimate was subsequently back-transformed to Pearson’s correlation coefficient (*r*) for interpretation.

### Model selection and heterogeneity

Given the inherent clinical heterogeneity across different surgical procedures (e.g., cardiovascular vs. non-cardiovascular) and the methodological variability in sampling protocols, a random-effects model (DerSimonian–Laird) was prespecified for all pooled analyses. This a priori choice was based on conceptual reasoning and was maintained regardless of the magnitude of statistical heterogeneity, as *I*² may be unreliable with few included studies. Statistical heterogeneity was quantified using *I*², with 25%, 50%, and 75% representing low, moderate, and high heterogeneity, respectively.

### Subgroup analyses and publication bias

Prespecified subgroup analyses compared studies involving cardiovascular surgery with those involving non-cardiovascular surgery. Additional exploratory subgroup analyses (e.g., on-pump vs. off-pump surgery) were performed for hypothesis-generating purposes. Publication bias was evaluated using visual inspection of funnel plots and Egger’s test [16,17]. When asymmetry was suggested, the trim-and-fill method was applied. All statistical analyses were conducted using Stata version 16.0 (StataCorp, College Station, TX, USA). A two-sided *p* < 0.05 was considered statistically significant.

## Results

### Search results

**Fig. 1:**
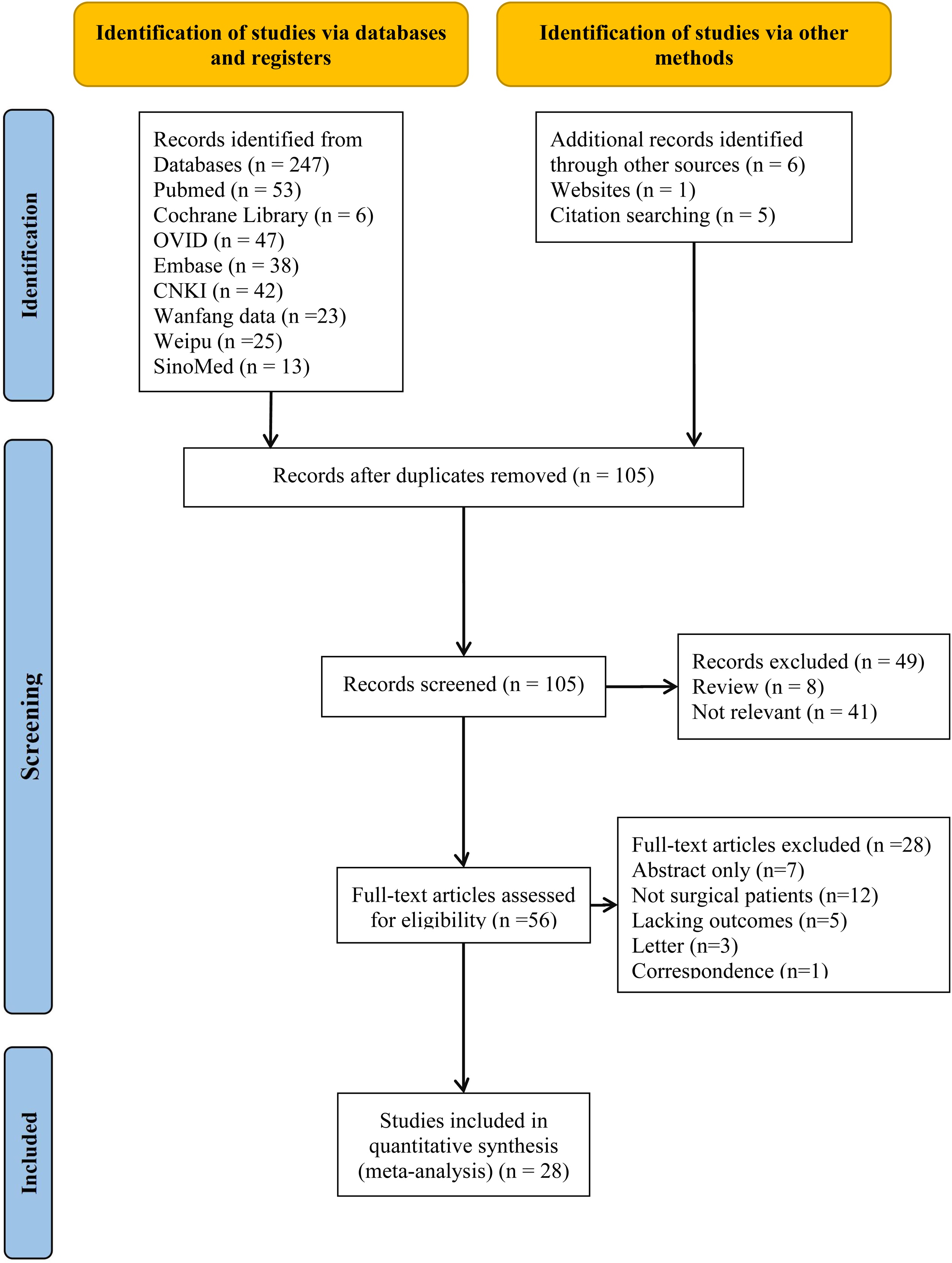
Flow diagram of studies included in the meta-analysis.

After removal of duplicates and screening of titles and abstracts, 28 studies [[7], [8], [9], [10], [11], [12],[18], [19], [20], [21], [22], [23], [24], [25], [26], [27], [28], [29], [30], [31], [32], [33], [34], [35], [36], [37], [38], [39]] comprising 1369 surgical patients met the inclusion criteria and were included in the meta-analysis (Fig. 1). The studies were conducted across multiple regions, including China (*n* = 6), Germany (*n* = 3), Egypt (*n* = 2), Belgium (*n* = 2), Saudi Arabia (*n* = 2), India (*n* = 2), and other countries (*n* = 11). Six studies were published in Chinese, one in Spanish, and the remainder in English. The main characteristics of the included studies are summarized in Table 1.

**Table 1:** Characteristics of included studies.

Study	Country	Population	Procedure	Patients No.	Sampling Site	Outcomes
Reinhart 1986 [18]	Germany	Adults	Aortobifemoral Bypass	105	SVC & PA	^a^
Nakayama 1996 [19]	Japan	Adults	Hepatectomy	25	SVC & PA	^a^
Zhang 1998 [20]	China	Pediatrics/Adults	CHD repair, VR	24	RA & PA	^a^
Turnaoğlu 2001 [21]	Turkey	Adults	Unspecified CS, abdominal aorta operations	32	SVC, RA & PA	^a,c^
Dueck 2005 [22]	Germany	Adults	Neurosurgical operation	70	SVC, RA & PA	^a,c^
Ramakrishna 2006 [23]	India	Adults	CABG/+ LV aneurysmorraphy, MVR	60	SVC & PA	^a,c^
Aggarwal 2007 [24]	India	Adults	AVR, CABG, MVR	20	RA & PA	^a^
Sander 2007 [25]	Germany	Adults	CABG	60	SVC & PA	^b,c^
Lorentzen 2008 [10]	Denmark	Adults	AVR, CABG, AVR + CABG, MVR	20	SVC & PA	^c^
Yazigi 2008 [11]	Lebanon	Adults	CABG	60	SVC & PA	^a,c^
el-Masry 2009 [7]	Egypt	Adults	OLT	50	SVC & PA	^a,c^
Sekkat 2009 [26]	Belgium	Adults	Unspecified CS	15	SVC & PA	^c^
Alshaer 2010 [27]	Saudi Arabia	Adults	CABG	34	RA & PA	^a^
Dahmani 2010 [28]	France	Adults	LT	30	RA & PA	^c^
Lequeux 2010 [29]	Belgium	Adults	Unspecified CS	15	SVC & PA	^c^
Soussi 2012 [12]	Tunisia	Adults	CABG	22	SVC & PA	^a,c^
Wu 201 [30]	China	Adults	OPCABG	37	SVC & PA	^a,c^
Li 2013 [31]	China	Adults	CABG, CHD repair, Aortic repair	50	SVC & PA	^a^
Elsherbeny 2014 [32]	Saudi Arabia	Adults	CABG, VR, CABG + VR	56	SVC & PA	^a,c^
Cavaliere 2014 [33]	Italy	Adults	CABG	30	SVC, RA & PA	^a,c^
Gasparovic 2014 [34]	Croatia	Adults	CABG, VR, CABG + VR, AVR	156	SVC & PA	^b,c^
Riva 2015 [35]	Uruguay	Adults	AVR, CABG, MVR	34	SVC & PA	^b,c^
Ali 2017 [36]	Egypt	Pediatrics	CHD repair (ASD, VSD, TOF, PAVC, SAM)	40	SVC & PA	^b,c^
Wang 2018 [9]	China	Adults	LTx	41	SVC & PA	^c^
Feng 2018 [37]	China	Adults	OPCABG	30	SVC & PA	^a,c^
Hu 2018 [38]	China	Pediatrics/Adults	CHD-PAH repair (VSD/+ASD, VSD/+PDA)	43	SVC & PA	^b,c^
Šoškić 2020 [8]	Serbia	Adults	AAAS	125	SVC & PA	^b^
Lanning 2022 [39]	Finland	Adults	CABG, AVR, MAP/MVR, Other procedures	85	RA & PA	^c^

Note. All studies included in this analysis were prospective cohort studies. Outcome measures were defined as follows: ^a^*r* values were correlation coefficients; ^b^*r* values were calculated based on *r*^2^ values; ^c^MD, mean difference, LOAs, limits of agreement.

Abbreviations: AVR, aortic valve replacement; ASD, atrial septal defect; AAAS, abdominal aortic aneurysm surgery; CHD, congenital heart disease; CHD-PAH, congenital heart disease with pulmonary arterial hypertension; CS, cardiac surgery; CABG, coronary artery bypass grafting; LT, Liver transplantation; LTx, lung transplantation; LV, left ventricle; MVR, mitral valve replacement; MAP, mitral annuloplasty; OPCAB, off-pump coronary artery bypass; OLT, orthotopic liver transplantation; PAVC, partial atriventricular canal; PDA, patent ductus arteriosus; PA, pulmonary arterial; RA, right atrium; SAM, subaortic membrane; SVC, superior vena cava; VR, valve replacement or repair; VSD, ventricular septal defect.

### Study characteristics and quality assessment

The included studies encompassed a broad patient population, ranging in age from 1 to 86 years. Most studies focused on adults. The primary clinical domain was cardiovascular surgery (23 of 28 studies), including cardiac procedures such as coronary artery bypass grafting (CABG), performed alone or combined with valvular surgery, repair of congenital heart disease, and aortic surgery. Major vascular procedures, including abdominal aortic aneurysm repair, were also represented. Both on-pump and off-pump techniques were represented. The remaining five studies involved major non-cardiovascular surgical procedures, including solid organ transplantation and neurosurgery. All surgeries were elective.

Methodological quality was assessed using the NOS, with studies rated as moderate to high quality, scoring between 4 and 7 points (Table 2).

**Table 2:** Newcastle–Ottawa Scale for observational cohort studies.

Study	Selection				Comparability	Outcome			Overall
	Representativeness of the exposed cohort	Selection of the non-exposed cohort	Ascertainment of exposure	Demonstration that outcome of interest was not present at start of study	Comparability of cohorts on the basis of the design or analysis	Assessment of outcome	Was follow-up long enough for outcomes to occur	Adequacy of follow-up of cohorts	
Reinhart 1986 [18]	★	☆	★	☆	☆☆	★	★	★	5
Nakayama 1996 [19]	★	☆	★	☆	★★	★	☆	☆	5
Zhang 1998 [20]	★	☆	★	☆	★★	★	☆	☆	5
Turnaoğlu 2001 [21]	★	☆	★	☆	★★	★	☆	☆	5
Dueck 2005 [22]	★	☆	★	☆	★★	★	☆	☆	5
Ramakrishna 2006 [23]	☆	☆	★	☆	★★	★	☆	☆	4
Aggarwal 2007 [24]	☆	☆	★	☆	★★	★	☆	★	5
Sander 2007 [25]	☆	☆	★	☆	★★	★	☆	★	5
Lorentzen 2008 [10]	☆	☆	★	☆	★★	★	☆	★	5
Yazigi 2008 [11]	☆	☆	★	☆	★★	★	☆	★	5
el-Masry 2009 [7]	☆	☆	★	☆	★★	★	☆	★	5
Sekkat 2009 [26]	★	☆	★	☆	★★	★	★	★	7
Alshaer 2010 [27]	★	☆	★	☆	★★	★	★	★	7
Dahmani 2010 [28]	★	☆	★	☆	★★	★	☆	☆	5
Lequeux 2010 [29]	★	☆	★	☆	★★	★	★	★	7
Soussi 2012 [12]	★	☆	★	★	☆☆	★	☆	☆	4
Wu 2012 [30]	★	☆	★	★	☆☆	★	★	★	6
Li 2013 [31]	★	★	★	☆	☆☆	★	★	★	6
Elsherbeny 2014 [32]	★	★	★	☆	☆☆	★	☆	★	5
Cavaliere 2014 [33]	★	★	★	★	★★	★	☆	☆	7
Gasparovic 2014 [34]	★	★	★	☆	★★	★	☆	★	7
Riva 2015 [35]	★	☆	★	☆	★★	☆	☆	☆	4
Ali 2017 [36]	★	☆	★	☆	★★	☆	☆	☆	4
Wang 2018 [9]	★	☆	★	☆	★★	☆	☆	☆	4
Feng 2018 [37]	★	☆	★	☆	★★	☆	☆	☆	4
Hu 2018 [38]	★	☆	★	☆	★★	☆	☆	☆	4
Šoškić 2020 [8]	★	☆	★	☆	★★	☆	☆	☆	4
Lanning 2022 [39]	★	☆	★	☆	★★	☆	☆	☆	4

☆, zero score; ★, one score; ★★, two scores.

Detailed patient demographics, surgical-anesthetic parameters, and perioperative data are provided in Supplementary Tables S1–S3. Specifications of the blood gas analyzers are summarized in Supplementary Table S4. Reporting and implementation of simultaneity for paired ScvO_2_ and SvO_2_ measurements varied across studies. Most studies merely stated that sampling was intended to be simultaneous. However, specific procedural details were generally not described (Supplementary Table S5).

### Bland–Altman and correlation analysis of ScvO_2_ and SvO_2_

**Fig. 2:**
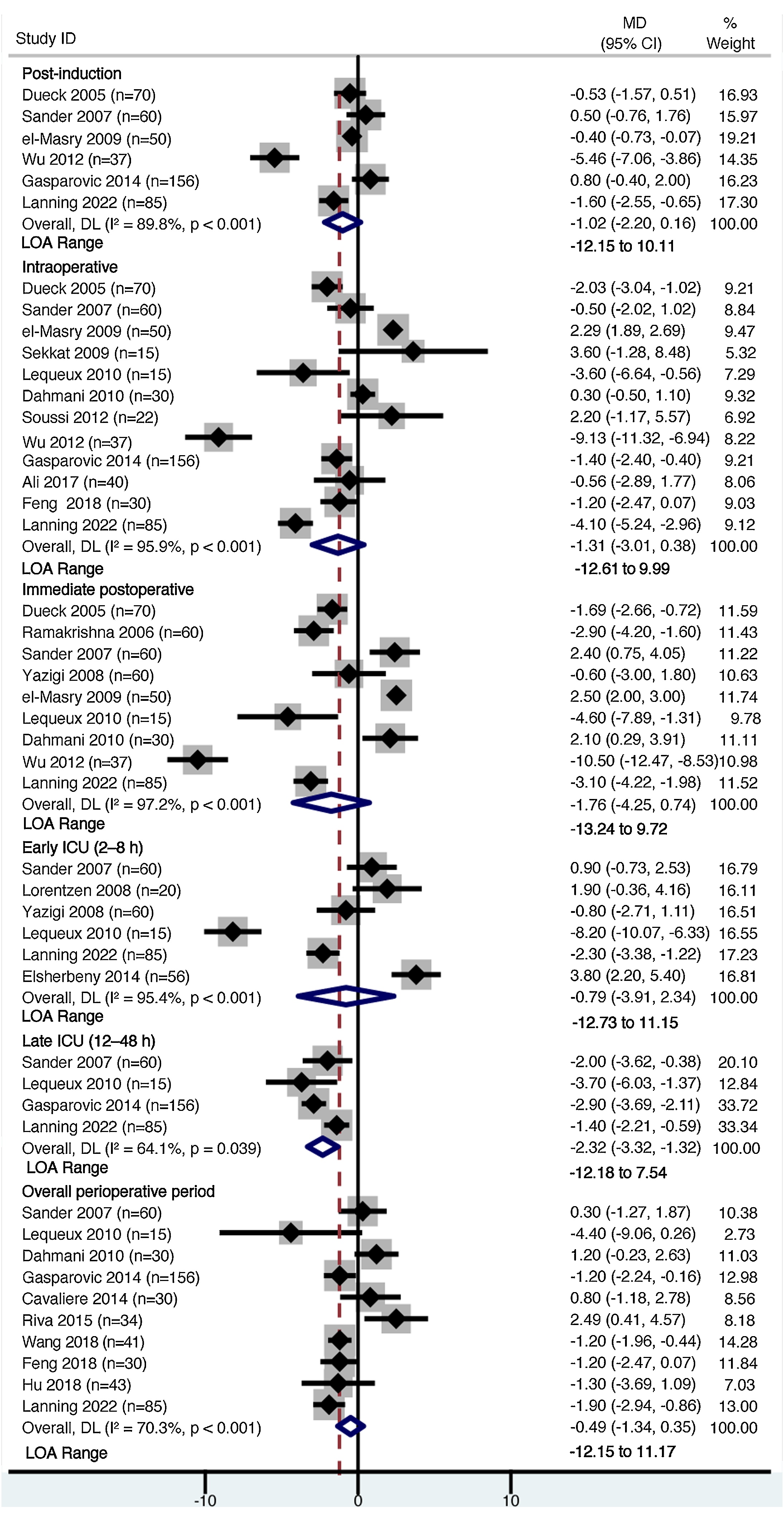
Forest plots of the pooled mean difference and limits of agreement between ScvO₂and SvO₂ across perioperative phases.

Bland–Altman meta-analysis revealed consistently small MDs but wide LOAs between ScvO_2_ and SvO_2_ across all perioperative phases (Fig. 2).

In the post-induction phase (6 studies, 458 patients), the pooled MD was −1.02% (95% CI −2.20 to 0.16), with corresponding LOA ranging from −12.15% to 10.11% (*I*² = 89.8%). During the intraoperative phase (12 studies, 610 patients), the pooled MD was −1.31% (95% CI −3.01 to 0.38), with LOA from −12.61% to 9.99% (*I*² = 95.9%). In the immediate postoperative phase (9 studies, 467 patients), the MD was −1.76% (95% CI −4.25 to 0.74), and LOA ranged from −13.24% to 9.72% (*I*² = 97.2%).

During the early intensive care unit (ICU) phase (6 studies, 296 patients), defined as the period from 2 to 8 h post-surgery, the pooled MD was −0.79% (95% CI −3.91 to 2.34), with LOA of −12.73% to 11.15% (*I*² = 95.4%). In the late ICU phase (12−48 h) (4 studies, 316 patients), the MD was −2.32% (95% CI − 3.32 to −1.32; *p* < 0.001), with LOA ranging from −12.18% to 7.54% (*I*² = 64.1%).

A separate meta-analysis aggregating perioperative data (10 studies, 524 patients) produced an overall MD of −0.49% (95% CI −1.34 to 0.35) and LOA from −12.15% to 11.17% (*I*² = 70.3%).

**Fig. 3:**
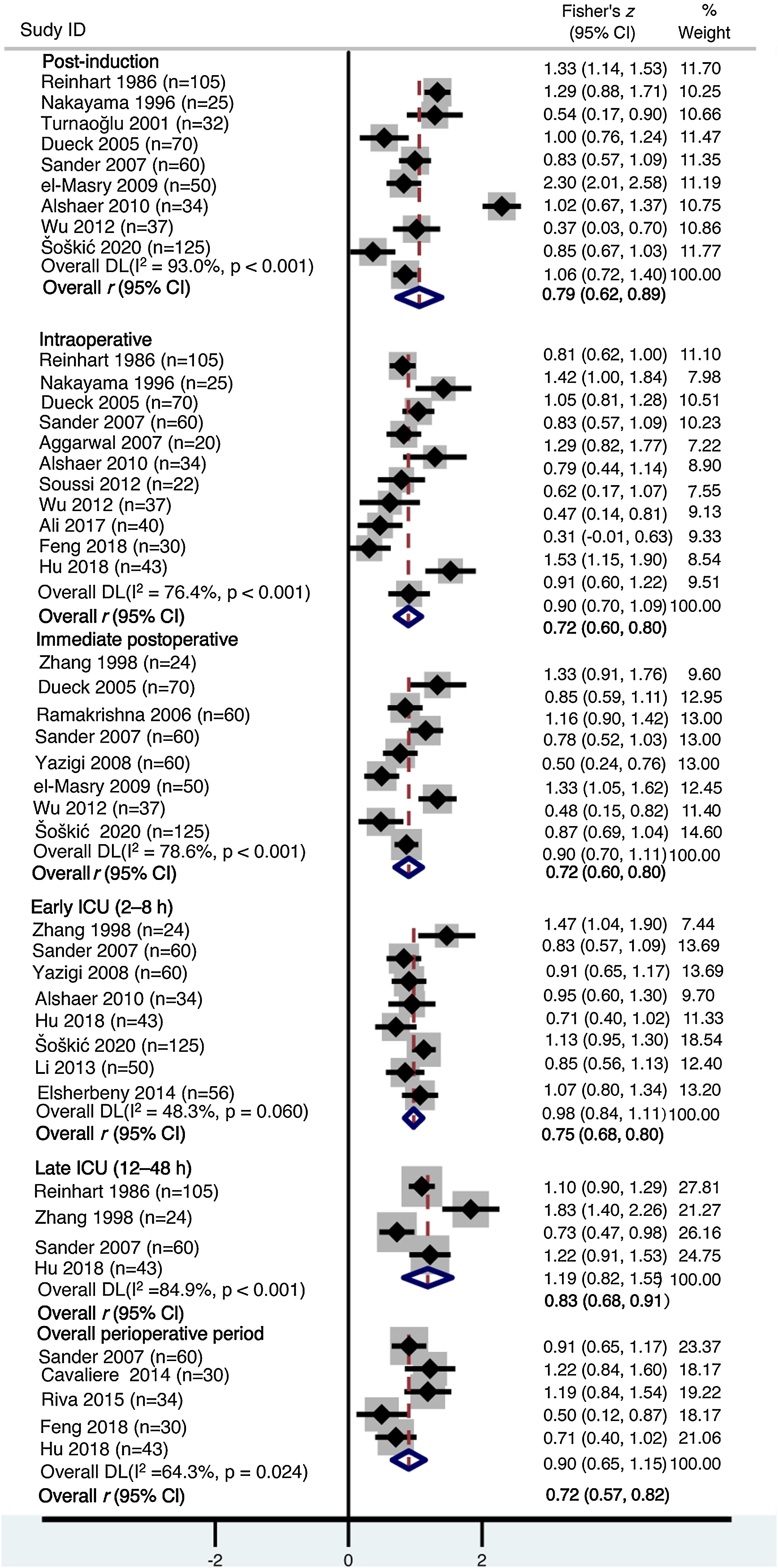
Forest plots of pooled Fisher’s *z*-transformed correlations between ScvO₂ and SvO₂ across perioperative phases.

Meta-analysis demonstrated a positive correlation between ScvO_2_ and SvO_2_ across all perioperative phases (Fig. 3). In the post-induction phase (9 studies, 538 patients), the pooled correlation coefficient was *r* = 0.79 (95% CI 0.62–0.89; *I*² = 93.0%). ScvO_2_ showed a correlation with SvO₂ intraoperatively (*r* = 0.72, 95% CI 0.60–0.80; *I*² = 76.4%; 11 studies, 486 patients) and in the immediate postoperative period (*r* = 0.72, 95% CI 0.60–0.80; *I*² = 78.6%; 8 studies, 486 patients). During ICU stay, ScvO_2_ and SvO_2_ remained correlated at the group level in both the early ICU phase (8 studies, 452 patients; *r* = 0.75, 95% CI 0.68–0.80; *I*² = 48.3%) and the late ICU phase (4 studies, 232 patients; *r* = 0.83, 95% CI 0.68–0.91; *I*² = 84.9%). Aggregated perioperative data (5 studies, 197 patients) showed an overall correlation of (*r* = 0.72, 95% CI 0.57–0.82; *I*² = 64.3%).

### Bland–Altman and correlation analysis of ΔScvO_2_ and ΔSvO_2_

Three studies (116 patients) reporting paired perioperative changes (ΔScvO_2_ and ΔSvO_2_) were included. The pooled MD was 0.07% (95% CI −1.59 to 1.73), with LOA ranging from −18.07% to 18.21%, reflecting limited agreement at the individual level (*I*² = 0%; Supplementary Fig. S1).

Four studies (186 patients) reporting correlations of changes were included. The pooled correlation coefficient was *r* = 0.66 (95% CI 0.52–0.77; *p* < 0.001), with moderate heterogeneity (*I*² = 53.9%; Supplementary Fig. S2).

### Subgroup analyses

Subgroup analyses (Supplementary Tables S6–S8) revealed superior ScvO_2_–SvO_2_ concordance in non-cardiovascular surgery compared with cardiovascular surgery, as reflected by narrower LOAs (−6.63% to 8.53% vs. −14.44% to 10.56%). A parallel improvement was observed in off-pump procedures, which exhibited a marked reduction in LOA width (LOA −9.81% to 6.25%) compared with on-pump procedures (LOA −14.23% to 11.97%).

### Sensitivity analysis and publication bias

Sensitivity analyses showed that exclusion of individual studies did not materially alter the overall pooled estimates or correlation coefficients (Supplementary Figs. S3–S4). Visual inspection of the funnel plot and Egger’s regression test suggested potential publication bias for the intraoperative MD (Supplementary Fig. S5). However, trim-and-fill analysis did not identify any missing studies, and the adjusted estimate reached statistical significance and was consistent with the fixed-effects model (Supplementary Fig. S6 and Table S9). Given the substantial between-study heterogeneity, the significant Egger’s test result may reflect heterogeneity rather than true publication bias. Although the adjusted intraoperative MD was statistically significant, the absolute difference was small and individual-level variation was considerable, and its clinical relevance is limited. For all other perioperative phases and pooled correlation coefficients, Egger’s test and funnel plots did not show evidence of significant publication bias, and no trim-and-fill adjustment was applied (Supplementary Fig. S7).

## Discussion

This systematic review and meta-analysis demonstrated a positive correlation between ScvO_2_ and SvO_2_ at the population level (*r* = 0.72–0.83). However, this association did not translate into individual-level agreement. Bland–Altman analysis revealed wide LOA (–12.15% to +11.17%), exceeded the prespecified ±5% margin, a commonly used but inherently context-dependent threshold for clinical acceptability. These findings indicate limited agreement at the individual level, suggesting that ScvO_2_ may not consistently approximate absolute SvO_2_ values across diverse perioperative conditions.

Previous studies in critically ill populations have shown that ScvO_2_ has moderate reliability for predicting SvO₂, with variability influenced by sampling site and sepsis [40]. However, these studies predominantly involved medical ICU patients, and their applicability to surgical populations remains uncertain. The present study specifically focused on perioperative patients and evaluated the relationship between ScvO_2_ and SvO_2_ across different surgical settings. By quantifying LOA at the individual level, this study provides additional evidence regarding the discrepancy between these two measures in surgical populations.

Notably, across perioperative phases, the LOA remained relatively stable yet consistently wide (approximately −12% to +10%), while MDs across studies showed a bidirectional distribution. These patterns suggest that the observed statistical features are more likely driven by combined anatomical, physiological, and methodological factors. SvO_2_ and ScvO_2_ originate from distinct venous drainage systems, representing different venous return compartments and sampling levels [6]. These structural differences introduce inherent measurement discordance but do not imply a consistent directional bias.

Perioperative dynamic changes in oxygen supply–demand balance may contribute to the observed variability, which may be further accentuated under conditions of marked hemodynamic stress, such as aortic cross-clamping. This balance is influenced by heterogeneous regional metabolic demands, redistribution of organ blood flow, and the use of vasoactive agents [[41], [42], [43]], all of which may, in different clinical contexts, affect the degree of concordance between SvO_2_ and ScvO_2_ as well as the characteristics of their bias. Between-study heterogeneity, including differences in patient populations, clinical settings, and study design, may also have influenced the estimated LOA. Collectively, these interpretations remain hypothesis-generating and should therefore be interpreted with caution, as the present study was not designed to establish mechanistic causality.

This study has several limitations. First, the included studies exhibited clinical and methodological heterogeneity, encompassing both cardiovascular and non-cardiovascular surgeries, which may have affected the consistency of the findings. Second, as a meta-analysis based on aggregated data, key physiological variables such as cardiac output, hemoglobin concentration, and oxygen supply–demand balance were insufficiently reported in the original studies. In addition, data on patient outcomes were limited, restricting further evaluation of clinically relevant endpoints. Third, averaging multiple measurements within the same perioperative phase may have reduced within-phase variability, potentially affecting agreement estimates. Moreover, most studies did not report strictly synchronized sampling protocols, and a lack of standardization in sampling and measurement methods may have introduced measurement variability. Finally, the predominance of single-center studies may limit the generalizability of the findings.

## Conclusions

In conclusion, ScvO_2_ is positively correlated with SvO_2_ in the perioperative period. However, wide individual-level LOA indicate that the two measurements are not interchangeable in surgical patients. LOA are narrower in non-cardiovascular and off-pump procedures but remain outside clinically acceptable thresholds. Future multicenter studies with standardized sampling protocols are needed to identify sources of individual-level variability and improve clinical interpretability.

## CRediT authorship contribution statement

YTY conceived and designed the study, developed the methodology, and supervised the entire project. LL and LXH were responsible for data acquisition, conducted formal analysis, and created visualizations. LL also contributed to data validation and drafted the original manuscript. YTY participated in the critical revision of the manuscript for important intellectual content. All authors reviewed and approved the final manuscript.

## Consent for publications

Not applicable.

## Funding

This work is supported by the Yunnan Provincial Clinical Medicine Research Special Program – (202405AJ310003).

## Ethics approval and consent to participate

Not applicable.

## Availability of data and materials

All data generated or analyzed during this study are available within the article and its supplementary materials.

## Declaration of competing interest

The authors declare that they have no competing interests.
