# Supplementary material for: Correlation between central venous oxygen saturation and mixed venous oxygen saturation in surgical patients: A systematic review and meta-analysis
Source: Ann Intensive Care. 2026 May 12;16:100076. doi: 10.1016/j.aicoj.2026.100076 (PMC13195361; doi:10.1016/j.aicoj.2026.100076)
Supplement: Supplementary file 1 [file mmc1.docx]

Supplemental Table S1. Characteristics of included patients

| **Study** | **Age** **(years)** | **Sex (M/F)** | **Weight (kg)** | **ASA** **Grade (I-V)** | **Other Reported Scores** | **LVEF (%)** |
| --- | --- | --- | --- | --- | --- | --- |
| Reinhart 1986^[18]^ | NR | NR | NR | NR | NR | NR |
| Nakayama 1996^[19]^ | NR | NR | NR | I~II | NR | NR |
| Zhang 1998^[20]^ | 33.2±11.6(7~53) | 16/8 | NR | NR | NR | NR |
| Turnaoğlu 2001^[21]^ | 55.5±14(15~82) | 18/14 | 50.4±7.4(37~65) | NR | APACHE II 15.9±3.9 | NR |
| Dueck 2005^[22]^ | 53.1±8.8(17~78) | 32/38 | 76.1±9.0(40~120) | Ⅰ~Ⅳ | NR | NR |
| Ramakrishna 2006^[23]^ | >18 | 55/5 | 55.6(33~73) | NR | NR | 38(20~50) |
| Aggarwal 2007^[24]^ | 48.95±14.13 | 12/8 | NR | NR | NR | NR |
| Sander 2007^[25]^ | 63(56-69) | 56/4 | 90(80~100) | NR | APACHE II 16(12~17) | NR |
| Lorentzen 2008^[10]^ | NR | 53/7 | NR | NR | APACHE II 9~14 | NR |
| Yazigi 2008^[11]^ | 68.0±6.8 (55~79) | 16/4 | **85.8****±15.7(**46~122) | NR | NR | NR |
| el-Masry 2009^[7]^ | 48.3±7.8 | 40/10 | 78.8**±**10.8 | III~IV | NR | NR |
| Sekkat 2009^[26]^ | 66 ±16 | 10/5 | 83**±**22 | NR | NR | NR |
| Alshaer 2010^[27]^ | 66±16 | 10/5 | 83**±**22 | NR | NR | 47.41±5.92 |
| Dahmani 2010^[28]^ | 48**±11** | 14/16 | NR | Ⅱ~Ⅲ | NR | NR |
| Lequeux 2010^[29]^ | 57.1±5.2 | 25/9 | NR | NR | NR | NR |
| Soussi 2012^[12]^ | 56.5±6.8 | 30/7 | 72.1±10.2 | II~III | NR | NR |
| Wu 2012^[30]^ | 61±7 | 20/2 | NR | NR | EuroSCORE 2±1% | 61±11 |
| Li 2013^[31]^ | 55.8±10.3(27~76) | 29/21 | NR | NR | NR | NR |
| Elsherbeny 2014^[32]^ | 67±9 | 30/26 | NR | NR | NR | NR |
| Cavaliere 2014^[33]^ | 68.9±9.2 | 26/4 | 76.9±11 | NR | NR | NR |
| Gasparovic 2014^[34]^ | 65±10 | 106/50 | NR | NR | EuroSCORE II 6.6 ± 11.0% | 63±14 |
| Riva 2015^[35]^ | 64±9 | 21/13 | NR | NR | EuroSCORE 7.4±4.9% | NR |
| Ali 2017^[36]^ | **4.54±3.06** | 22/18 | 21.52±10.61 | NR | NR | NR |
| Wang 2018^[9]^ | 31.0±11.0 | 15/28 | NR | NR | NR | NR |
| Feng 2018^[37]^ | 53.8±15.4(19~76) | 29/12 | 55.2±13.0 (35~86) | Ⅲ~Ⅳ | NR | 53±14 |
| Hu 2018^[38]^ | 68.3±5.5(65~80) | 18/12 | NR | NR | NR | NR |
| Šoškić 2020^[8]^ | 66.39±6.49(49~86) | 108/17 | NR | NR | NR | NR |
| Lanning 2022^[39]^ | 65(59~71) | 70/15 | 82 (71~91) | NR | NYHA class III | >50: 64; 31~50: 20; 21~30: 1 |

Abbreviations: ASA, American Society of Anesthesiologists Physical Status Classification; APACHE, Acute Physiology and Chronic Health Evaluation; EuroSCORE, European System for Cardiac Operative Risk Evaluation; NYHA, New York Heart Association; LVEF, left ventricular ejection fraction; NR, not reported.
