# Supplementary material for: Correlation between central venous oxygen saturation and mixed venous oxygen saturation in surgical patients: A systematic review and meta-analysis
Source: Ann Intensive Care. 2026 May 12;16:100076. doi: 10.1016/j.aicoj.2026.100076 (PMC13195361; doi:10.1016/j.aicoj.2026.100076)
Supplement: Supplementary file 2 [file mmc2.docx]

**Supplemental Table S2.** **Surgical details of the included studies**

| **Study** | **Procedure** | **CPB Used** | **CPB Duration (min)** | **Aortic Clamp Duration (min)** | **Operative Duration (min)** |
| --- | --- | --- | --- | --- | --- |
| Reinhart 1986^[18]^ | **Aortobifemoral Bypass** | Off-Pump | NA | NA | NR |
| Nakayama 1996^[19]^ | Hepatectomy | Off-Pump | NA | NA | NR |
| Zhang 1998^[20]^ | CHD repair, VR | On-Pump | NR | NR | NR |
| Turnaoğlu 2001^[21]^ | Unspecified CS, abdominal aorta operations | NR | NR | NR | NR |
| Dueck 2005^[22]^ | Neurosurgical operation | Off-Pump | NA | NA | NR |
| Ramakrishna 2006^[23]^ | CABG/+ LV aneurysmorraphy, MVR | On-Pump | NR | NR | NR |
| Aggarwal 2007^[24]^ | AVR, CABG, MVR | On-Pump | NR | NR | NR |
| Sander 2007^[25]^ | CABG | On-Pump | 73(59~82) | 45(36~54) | 200 (173~225) |
| Lorentzen 2008^[10]^ | CABG | On-Pump | NR | NR | NR |
| Yazigi 2008^[11]^ | AVR, CABG, AVR+CABG, MVR | On-Pump | NR | NR | NR |
| el-Masry 2009^[7]^ | OLT | Off-Pump | NA | NA | NR |
| Sekkat 2009^[26]^ | Unspecified CS | On-Pump | 120(46) | 80(34) | 245(71) |
| Alshaer 2010^[27]^ | CABG | On-Pump | 120±46 | 80±34 | 245±71 |
| Dahmani 2010^[28]^ | LT | Off-Pump | NA | NA | 564±154 |
| Lequeux 2010^[29]^ | Unspecified CS | On-Pump | 109.40±18.51 | 0 | NR |
| Soussi 2012^[12]^ | CABG | Off-Pump | NA | NA | NR |
| Wu 2012^[30]^ | OPCABG | On-Pump | 118±52 | 78±30 | NR |
| Li 2013^[31]^ | **CABG, CHD** **repair,** Aortic repair | On-Pump | NR | NR | NR |
| Elsherbeny 2014^[32]^ | CABG, VR, CABG+VR | On-Pump | NR | NR | NR |
| Cavaliere 2014^[33]^ | CABG | On-Pump | NR | NR | NR |
| Gasparovic 2014^[34]^ | CABG, VR, CABG+VR, AVR | On-Pump | 115 ± 58 | 81±40 | NR |
| Riva 2015^[35]^ | AVR, CABG, MVR | On-Pump | 143±8 | 78±28 | NR |
| Ali 2017^[36]^ | CHD **repair (ASD, VSD, TOF,** PAVC, SAM**)** | On-Pump | NR | 79.60(37.22) | NR |
| Wang 2018^[9]^ | **LTx** | Off-Pump | NA | NA | NR |
| Feng 2018^[37]^ | OPCABG | Off-Pump | NA | NA | NR |
| Hu 2018^[38]^ | CHD-PAH repair (VSD/+ASD, VSD/+PDA) | On-Pump | 56.0±15.0 | 32.3±12.4 | 186.0±39.0 |
| Šoškić 2020^[8]^ | AAAS | Off-Pump | NA | NA | NR |
| Lanning 2022^[39]^ | CABG, AVR, MAP/MVR, Other procedures | On-Pump | 375(322~424) | NR | NR |

Abbreviations: AVR, aortic valve replacement; ASD, atrial septal defect; AAAS, abdominal aortic aneurysm surgery; CHD, congenital heart disease; CHD-PAH, congenital heart disease with pulmonary arterial hypertension; CS, cardiac surgery; CABG, coronary artery bypass grafting; CPB, cardiopulmonary bypass. LT, Liver transplantation; LTx, lung transplantation; LV, left ventricle; MVR, mitral valve replacement; MAP, mitral annuloplasty; NA, not applicable; NR, not reported; OPCAB, off-pump coronary artery bypass; OLT, orthotopic liver transplantation; Off-Pump, off-cardiopulmonary bypass; On-Pump, on-cardiopulmonary bypass; PAVC, partial atriventricular canal; PDA, patent ductus arteriosus; SAM, subaortic membrane; VR, valve replacement or repair; VSD, ventricular septal defect.
