# Supplementary material for: Correlation between central venous oxygen saturation and mixed venous oxygen saturation in surgical patients: A systematic review and meta-analysis
Source: Ann Intensive Care. 2026 May 12;16:100076. doi: 10.1016/j.aicoj.2026.100076 (PMC13195361; doi:10.1016/j.aicoj.2026.100076)
Supplement: Supplementary file 3 [file mmc3.docx]

**Supplemental Table S3.** Anesthetic agents and FiO₂ in included studies

| **Study** | **Anesthetic Agents** |  | | **FiO₂** |
| --- | --- | --- | --- | --- |
| Reinhart 1986^[18]^ | Halothane, NLA, TEA | | | O₂/N₂O (1:2) |
| Nakayama 1996^[19]^ | Thiamylal, vecuronium, isoflurane | | | O₂/N₂O |
| Zhang 1998^[20]^ | NR | | | 50% |
| Turnaoğlu 2001^[21]^ | Fentanyl, isoflurane | | | 54%±23% |
| Dueck 2005^[22]^ | Fentanyl, midazolam | | | NR |
| Ramakrishna 2006^[23]^ | NR | | | 38% |
| Aggarwal 2007^[24]^ | Etomidate, fentanyl, pancuronium, midazolam, isoflurane | | | 50% |
| Sander 2007^[25]^ | Phenargan, morphine, thiopentone, rocuronium, fentanyl, pancuronium, midazolam, isoflurane | | | NR |
| Lorentzen 2008^[10]^ | Etomidate, pancuronium, midazolam, fentanyl, isoflurane | | | NR |
| Yazigi 2008^[11]^ | Diazepam, sufentanil, propofol, pancuronium | | | air/O₂ |
| el-Masry 2009^[7]^ | Propofol, fentanyl, atracurium, sevoflurane | | | NR |
| Sekkat 2009^[26]^ | propofol, remifentanil, cisatracurium | | | NR |
| Alshaer 2010^[27]^ | Midazolam, propofol, remifentanil, cisatracurium + SA (sufentanil and morphine) | | | air/O₂ |
| Dahmani 2010^[28]^ | Lorazepam, morphine, sufentanil, midazolam, rocuronium, sevoflurane | | | 40% |
| Lequeux 2010^[29]^ | Propofol, succinylcholine, desflurane, sufentanil, atracurium | | | 50% |
| Soussi 2012^[12]^ | Morphine, scopolamine, midazolam, rocuronium, sufentanil, propofol, Pipecuronium | | | 73% |
| Wu 2012^[30]^ | Hydroxyzine, propofol, remifentanil, cisatracurium | | | air/O₂ |
| Li 2013^[31]^ | NR | | | NR |
| Elsherbeny 2014^[32]^ | NR | | | NR |
| Cavaliere 2014^[33]^ | Midazolam, fentanyl, pancuronium, sevoflurane | | | 50% |
| Gasparovic 2014^[34]^ | NR | | | NR |
| Riva 2015^[35]^ | NR | | | NR |
| Ali 2017^[36]^ | NR | | | NR |
| Wang 2018^[9]^ | NR | | 70~100% | |
| Feng 2018^[37]^ | Midazolam, etomidate, sufentanil, cisatracurium, propofol, remifentanil, dexmedetomidine, sevoflurane | | | NR |
| Hu 2018^[38]^ | Sufentanil, etomidate, rocuronium | | | 50% |
| Šoškić 2020^[8]^ | Combined epidural/GA: midazolam, rocuronium, levobupivacain, sevoflurane | | | 50% |
| Lanning 2022^[39]^ | Propofol, remifentanil, rocuronium, sevoflurane | | | NR |

Abbreviations: FiO₂, inspired oxygen fraction; NR, not reported; NLA, neuroleptanalgesia; PA, pulmonary artery; TEA, thoracic epidural analgesia.
