# Supplementary material for: Correlation between central venous oxygen saturation and mixed venous oxygen saturation in surgical patients: A systematic review and meta-analysis
Source: Ann Intensive Care. 2026 May 12;16:100076. doi: 10.1016/j.aicoj.2026.100076 (PMC13195361; doi:10.1016/j.aicoj.2026.100076)
Supplement: Supplementary file 4 [file mmc4.docx]

Supplement Table S4. Blood gas analysis and advanced monitoring equipment in the included studies

| **Study** | **Measurement Method** | **Device (Model/Manufacturer)** |
| --- | --- | --- |
| Reinhart 1986^[18]^ | Intermittent blood gas analysis | BGA: IL-282 Co-Oximeter (Instrumentation Laboratory); PAC: Cordis introducer sheath (7.5F) |
| Nakayama 1996^[19]^ | Intermittent blood gas analysis | BGA: Corning 2500 Co-Oximeter (Ciba-Corning); PAC: 7.0F thermodilution flow-directed catheter (Baxter Health Care) |
| Zhang 1998^[20]^ | Intermittent blood gas analysis | BGA: HP782050 (Model not specified); PAC: Not specified |
| Turnaoğlu 2001^[21]^ | Intermittent blood gas and co-oximetry analysis | BGA: ABL 505 and OSM3 Co-Oximeter (Radiometer, Copenhagen, Denmark); PAC: 7F (Abbott Systems, Sligo, Ireland) |
| Dueck 2005^[22]^ | Intermittent co-oximetric analysis | Co-oximeter: OSM 3 Hemoximeter (Radiometer, Copenhagen, Denmark); CVC: Multiorificed CVC (Vygon GmbH, Aachen, Germany); PAC: OptiQ® (Abbott, Chicago, IL, USA) |
| Ramakrishna 2006^[23]^ | Intermittent blood gas analysis | BGA: Bayer Health Care (Model not specified); CVC: Multiorificed 7.5F catheter (Model not specified); PAC: Introduced via 8.5F sheath |
| Aggarwal 2007^[24]^ | Intermittent blood gas analysis | BGA: Model not specified; CVC: High Flow catheter (Edwards Lifesciences LLC, Irvine, CA, USA); PAC: 7.5F (Edwards Lifesciences LLC, Irvine, CA, USA) |
| Sander 2007^[25]^ | Intermittent blood gas analysis | BGA: ABL-700 series (Radiometer, Copenhagen, Denmark); PAC: Arrow (Reading, PA, USA) |
| Lorentzen 2008^[10]^ | Intermittent blood gas and co-oximetry analysis | BGA: Gem Premier 3000 (Instrumentation Laboratory, Lexington, MA, USA);  CVC: 7F (Arrow-Howes, Reading, PA, USA); PAC: 7.5F (Edwards Lifesciences, Irvine, CA, USA); |
| Yazigi 2008^[11]^ | Intermittent central venous blood gas analysis + continuous SvO₂ monitoring | BGA: System 610/Series 77 (Radiometers ABL, Copenhagen, Denmark);  SvO₂ monitor: Vigilance monitor (Edwards Lifesciences, Irvine, CA, USA);  PAC: 744 HF75 (Edwards Lifesciences, Irvine, CA, USA) |
| el-Masry 2009^[7]^ | Intermittent blood gas and co-oximetry analysis (simultaneous sampling from CVC and PAC) | BGA: ABL 700 co-oximeter (Radiometer, Copenhagen, Denmark); CVC: 7-Fr triple lumen CVP catheter (Arrow International Inc, Reading, PA, USA); PAC: OPTIQ SVO2/CCO (Abbott Laboratories, North Chicago, IL, USA) |
| Sekkat 2009^[26]^ | Continuous fiberoptic monitoring + intermittent blood gas analysis for calibration | BGA: Model not specified; CVC: CeVOX fiberoptic catheter (Pulsion Medical Systems)  PAC: Edwards Lifesciences (Model not specified); |
| Alshaer 2010^[27]^ | Intermittent blood gas analysis | BGA: QS 50 (Radiometer, Copenhagen, Denmark); PAC: 7.5F (Edwards Lifesciences, Irvine, CA, USA) |
| Dahmani 2010^[28]^ | Intermittent blood gas analysis | BGA: Model not specified; PAC: 7.5 Fr three-lumen PAC (Edwards Lifesciences) |
| Lequeux 2010^[29]^ | Continuous fiberoptic monitoring | Monitor for SvO₂: Vigilance monitor (Edwards Lifesciences) + Multi Data Logger software.; Monitor for ScvO₂: CeVOX monitor (Pulsion Medical Systems) + CeVOX Win software; CVC: 7F three-lumen catheter (Arrow International Inc.) Three-lumen catheter (Arrow International Inc.); Central venous oximetry catheter: CeVOX fiber-optic catheter (Pulsion Medical Systems); PAC: Edwards Lifesciences (Model not specified) |
| Soussi 2012^[12]^ | Intermittent blood gas analysis | BGA for SvO₂: Synthesis™ 20 (Instrumentation Laboratory, Lexington, KY, USA); Monitor for ScvO₂: Vigilance™ monitor (Edwards Lifescience); CVC: Edwards Lifescience X3820HS (Irvine, CA, USA) |
| Wu 2012^[30]^ | Intermittent blood gas analysis (for both SvO₂ & ScvO₂) | BGA: Model not specified; CVC: PreSep X3820HS (Edwards Lifesciences)  PAC: CCO/CEDV/SvO₂ 774HF75 (Edwards Lifesciences); |
| Li 2013^[31]^ | Intermittent blood gas analysis | NR |
| Elsherbeny 2014^[32]^ | Intermittent blood gas analysis | NR |
| Cavaliere 2014^[33]^ | Intermittent co-oximetry analysis | Co-oximeter: Stat Profile pHOx Ultra Analyzer (Nova Biomedical Co., USA); CVC: three-lumen 7 Fr catheter (Arrow International Inc, USA); PAC: 7 Fr PAC (Edwards Lifescience LLC, USA); Introducer: 8 Fr introducer (Edwards Lifescience LLC, USA) |
| Gasparovic 2014^[34]^ | Intermittent blood gas analysis | BGA: RAPIDlab 1265 (Siemens, Muenchen, Germany); PAC: Argon Medical Devices (Singapore) |
| Riva 2015^[35]^ | Intermittent blood gas analysis | BGA: ABL® 700 series (Radiometer, Copenhagen, Denmark); PAC: 7.5 G CAP (Biosensors International Pte Ltd, Singapore) |
| Ali 2017^[36]^ | Intermittent blood gas analysis | BGA: EDAN i15-FCC (EDAN, China); CVC: Double-lumen 5 Fr ARROW Blue Catheters (B. Braun Melsungen AG, Germany) |
| Wang 2018^[9]^ | Intermittent pulmonary artery blood gas analysis + continuous central venous oxygen saturation monitoring | BGA: Model not specified; Continuous monitoring system: Edwards central venous catheter connected to a Vigileo monitor |
| Feng 2018^[37]^ | Intermittent blood gas analysis | BGA: GEM Premier 3000 (Instrumentation Laboratory);  CVC: Triple-lumen catheter  PAC: CCO/SvO2 774HF75 (Edwards Lifesciences) connected to Vigilance II monitor; |
| Hu 2018^[38]^ | Intermittent blood gas analysis | BGA: ABL-700 series (Radiometer, Denmark); CVC: SCW-CVCP-2 (Shenzhen Yixinda Medical New Technology Co., Ltd., China); PAC: 774F75 (Edwards Lifescience LLC, USA) |
| Šoškić 2020^[8]^ | Intermittent blood gas analysis | BGA: Radiometer ABL 90 flex (Radiometer); CVC: Arrow (Model not specified); PAC: Swan-Ganz catheter, Arrow, 7F (Arrow International) |
| Lanning 2022^[39]^ | Intermittent blood gas analysis | BGA: GEM Premier 4000; PAC: Criticath SP5507U TD |

Abbreviations: BGA, blood gas analyzer; CVC, central venous catheter; NR, not reported; PAC, pulmonary artery catheter.
