# Supplementary material for: Correlation between central venous oxygen saturation and mixed venous oxygen saturation in surgical patients: A systematic review and meta-analysis
Source: Ann Intensive Care. 2026 May 12;16:100076. doi: 10.1016/j.aicoj.2026.100076 (PMC13195361; doi:10.1016/j.aicoj.2026.100076)
Supplement: Supplementary file 5 [file mmc5.docx]

**Supplemental Table S5.** Reporting of simultaneity for paired ScvO₂ and SvO₂ measurements

| **Study** | **Sampling timing description** | **Implementation details** |
| --- | --- | --- |
| Reinhart 1986^[18]^ | Simultaneous | NR |
| Nakayama 1996^[19]^ | Simultaneous | NR |
| Zhang 1998^[20]^ | Separately collected | NR |
| Turnaoğlu 2001^[21]^ | Rapid succession | RV sample → catheter withdrawn → RA sample → catheter advanced → PA sample → arterial sample → CV sample (from introducer sideport) |
| Dueck 2005^[22]^ | Simultaneous | NR |
| Ramakrishna 2006^[23]^ | Simultaneous | NR |
| Aggarwal 2007^[24]^ | Simultaneous | NR |
| Sander 2007^[25]^ | Simultaneous | NR |
| Lorentzen 2008^[10]^ | Simultaneous | ScvO₂ from CVC blood draw; SvO₂ from Vigilance monitor, obtained simultaneously |
| Yazigi 2008^[11]^ | Simultaneous | All samples withdrawn from PA and CVC over 30 seconds using low-negative-pressure |
| el-Masry 2009^[7]^ | Simultaneous | Within 30 seconds |
| Sekkat 2009^[26]^ | Simultaneous | SvO₂ and ScvO₂ continuously monitored and recorded every minute via computer software for 24 hours |
| Alshaer 2010^[27]^ | Simultaneous | NR |
| Dahmani 2010^[28]^ | Simultaneous | NR |
| Lequeux 2010^[29]^ | Simultaneous | SvO₂ and ScvO₂ continuously monitored and recorded every minute via computer software for 24 hours |
| Soussi 2012^[12]^ | Simultaneous | NR |
| Wu 2012^[30]^ | NR | NR |
| Li 2013^[31]^ | Separately collected | NR |
| Elsherbeny 2014^[32]^ | Simultaneous | NR |
| Cavaliere 2014^[33]^ | Simultaneous | within 60 seconds |
| Gasparovic 2014^[34]^ | Simultaneous | NR |
| Riva 2015^[35]^ | Simultaneous | NR |
| Ali 2017^[36]^ | Simultaneous | NR |
| Wang 2018^[9]^ | Simultaneous | NR |
| Feng 2018^[37]^ | Simultaneous | NR |
| Hu 2018^[38]^ | Simultaneous | NR |
| Šoškić 2020^[8]^ | Simultaneous | NR |
| Lanning 2022^[39]^ | Successive | Sequential sampling |

Abbreviations: CV, central venous; NR, not reported; PA, pulmonary artery, RA, Right Atrium; RV, Right Ventricle.
