# Supplementary material for: Correlation between central venous oxygen saturation and mixed venous oxygen saturation in surgical patients: A systematic review and meta-analysis
Source: Ann Intensive Care. 2026 May 12;16:100076. doi: 10.1016/j.aicoj.2026.100076 (PMC13195361; doi:10.1016/j.aicoj.2026.100076)
Supplement: Supplementary file 6 [file mmc6.docx]

Supplemental Table S6. ScvO₂–SvO₂ correlation and mean difference: cardiovascular versus non-cardiovascular surgical patients

| **Outcomes** | **Trials** | | | **Total (n)** | **Pooled value** | **95%CI** | | ***I*^2^** | **Heterogeneity *p*** | **Model** | **Overall Effect *p*** |
| --- | --- | --- | --- | --- | --- | --- | --- | --- | --- | --- | --- |
| **MD** |  | | |  |  |  |  | |  |  |  |
| PostInd | |  | |  |  |  | |  |  |  |  |
| CVS | 4 | | | 338 | -1.40 | -3.77, 0.98 | | 93.4% | <0.001 | REM | 0.25 |
| NCVS | 2 | | | 120 | -0.41 | -0.72, -0.10 | | 0% | 0.82 | FEM | 0.01 |
| Total | 6 | | | 458 | -1.02 | -2.20, 0.16 | | 89.8% | <0.001 | REM | 0.09 |
| Intraop |  | | |  |  |  | |  |  |  |  |
| CVS | 9 | | | 460 | -1.94 | -3.74, -0.15 | | 89.1% | <0.001 | REM | 0.03 |
| NCVS | 3 | | | 150 | 0.22 | -2.22, 2.66 | | 97.1% | <0.001 | REM | 0.86 |
| Total | 12 | | | 610 | -1.31 | -3.01, 0.38 | | 95.9% | <0.001 | REM | 0.13 |
| Immed PO |  | | |  |  |  | |  |  |  |  |
| CVS | 6 | | | 317 | -3.09 | -6.37, -0.01 | | 95.2% | <0.001 | REM | 0.05 |
| NCVS | 3 | | | 150 | 0.95 | -2.03, 3.94 | | 96.4% | <0.001 | REM | 0.53 |
| Total | 9 | | | 467 | -1.76 | -4.27, 0.75 | | 97.2% | <0.001 | REM | 0.17 |
| Overall Periop |  | | |  |  |  | |  |  |  |  |
| CVS | 9 | | | 494 | -0.72 | -1.53, 0.09 | | 63.6% | 0.005 | REM | 0.08 |
| NCVS | 1 | | | 30 | 1.20 | -0.23, 2.63 | | NA | NA | NA | 0.10 |
| Total | 10 | | | 524 | -0.49 | -1.34, 0.35 | | 70.3% | <0.001 | REM | 0.25 |
|  |  | | |  |  |  | |  |  |  |  |
| ***r*** |  | | |  |  |  | |  |  |  |  |
| PostInd | | |  |  |  |  | |  |  |  |  |
| CVS | 6 | | | 393 | 0.69 | 0.51, 0.80 | | 85.1% | <0.001 | REM | <0.001 |
| NCVS | 3 | | | 145 | 0.91 | 0.58, 0.98 | | 95.8% | <0.001 | REM | <0.001 |
| Total | 9 | | | 538 | 0.79 | 0.62, 0.89 | | 93.0% | <0.001 | REM | <0.001 |
| Intraop |  | | |  |  |  | |  |  |  |  |
| CVS | 9 | | | 391 | 0.68 | 0.54, 0.78 | | 75.1% | <0.001 | REM | <0.001 |
| NCVS | 2 | | | 95 | 0.83 | 0.68, 0.91 | | 57.4% | 0.125 | REM | <0.001 |
| Total | 11 | | | 486 | 0.72 | 0.60, 0.80 | | 76.4% | <0.001 | REM | <0.001 |
| Immed PO |  | | |  |  |  | |  |  |  |  |
| CVS | 8 | | | 366 | 0.69 | 0.54, 0.79 | | 77.4% | <0.001 | REM | <0.001 |
| NCVS | 2 | | | 120 | 0.79 | 0.54, 0.92 | | 84.6% | 0.011 | REM | <0.001 |
| Total | 10 | | | 486 | 0.72 | 0.60, 0.80 | | 78.6% | <0.001 | REM | <0.001 |

Abbreviations: CVS, cardiovascular surgery; Intraop, intraoperative period; Immed PO, immediate postoperative period; MD, mean difference; *r*, correlation coefficient; NCVS, non-cardiovascular surgery; NA, not applicable; Overall Periop, overall perioperative period; PostInd, post-induction period; REM, random effect model; 95% CI, 95% confidence interval.
