# Supplementary material for: Correlation between central venous oxygen saturation and mixed venous oxygen saturation in surgical patients: A systematic review and meta-analysis
Source: Ann Intensive Care. 2026 May 12;16:100076. doi: 10.1016/j.aicoj.2026.100076 (PMC13195361; doi:10.1016/j.aicoj.2026.100076)
Supplement: Supplementary file 7 [file mmc7.docx]

Supplemental Table S7. ScvO₂–SvO₂ correlation and mean difference: on- versus off-pump patients

| **Outcomes** | **Trials** | | **Total(n)** | **Pooled value** | **95%CI** | ***I*^2^** | **Heterogeneity *p*** | **Model** | **Overall Effect *p*** |
| --- | --- | --- | --- | --- | --- | --- | --- | --- | --- |
| **MD** |  | |  |  |  |  |  |  |  |
| PostInd |  | |  |  |  |  |  |  |  |
| On-Pump | 3 | | 301 | -0.14 | -1.73, 1.45 | 83.1% | 0.003 | REM | 0.86 |
| Off-Pump | 3 | | 157 | -2.00 | -4.30, 0.31 | 94.6% | <0.001 | REM | 0.08 |
| Total | 6 | | 458 | -1.02 | -2.20, 0.16 | 89.8% | <0.001 | REM | 0.09 |
| Intraop |  | |  |  |  |  |  |  |  |
| On-Pump | 7 | | 393 | -1.13 | -2.77, 0.52 | 80.9% | <0.001 | REM | 0.18 |
| Off-Pump | 5 | | 217 | -1.78 | -4.39, 0.83 | 97.7% | <0.001 | REM | 0.18 |
| Total | 12 | | 610 | -1.31 | -3.01, 0.38 | 95.9% | <0.001 | REM | 0.13 |
| Immed PO |  | |  |  |  |  |  |  |  |
| On-Pump | 5 | | 280 | -1.67 | -3.97, 0.63 | 89.1% | <0.001 | REM | 0.16 |
| Off-Pump | 4 | | 187 | -1.84 | -6.25, 2.58 | 98.5% | <0.001 | REM | 0.42 |
| Total | 9 | | 467 | -1.76 | -4.25, 0.74 | 97.2% | <0.001 | REM | 0.17 |
| Overall Periop |  | |  |  |  |  |  |  |  |
| On-Pump | 7 | | 423 | -0.85 | -2.60, 0.91 | 86.7% | 0.021 | REM | 0.34 |
| Off-Pump | 3 | | 101 | -0.49 | -1.87, 0.90 | 77.6% | <0.0001 | REM | 0.49 |
| Total | 10 | | 524 | -0.70 | -1.84, 0.43 | 84.0% | <0.0001 | REM | 0.23 |
|  |  | |  |  |  |  |  |  |  |
| ***r*** |  | |  |  |  |  |  |  |  |
| PostInd | |  |  |  |  |  |  |  |  |
| On-Pump | 2 | | 94 | 0.72 | 0.60, 0.80 | 0% | 0.39 | FEM | <0.001 |
| Off-Pump | 6 | | 406 | 0.83 | 0.62, 0.93 | 95.0% | <0.001 | REM | <0.001 |
| Total | 8 | | 500 | 0.81 | 0.64, 0.90 | 93.3% | <0.001 | REM | <0.001 |
| Intraop |  | |  |  |  |  |  |  |  |
| On-Pump | 6 | | 219 | 0.65 | 0.49, 0.77 | 64.0% | 0.016 | REM | <0.001 |
| Off-Pump | 5 | | 267 | 0.78 | 0.61, 0.88 | 83.5% | <0.001 | REM | <0.001 |
| Total | 11 | | 486 | 0.72 | 0.60, 0.80 | 76.4% | <0.001 | REM | <0.001 |
| Immed PO |  | |  |  |  |  |  |  |  |
| On-Pump | 4 | | 204 | 0.71 | 0.58, 0.76 | 83.0% | 0.003 | REM | <0.001 |
| Off-Pump | 4 | | 282 | 0.75 | 0.59, 0.86 | 80.0% | 0.009 | REM | <0.001 |
| Total | 8 | | 486 | 0.72 | 0.60, 0.80 | 78.6% | <0.001 | REM | <0.001 |
| Early ICU (2–8 h) |  | |  |  |  |  |  |  |  |
| On-Pump | 6 | | 327 | 0.74 | 0.59, 0.83 | 65.2% | 0.035 | REM | <0.001 |
| Off-Pump | 1 | | 125 | 0.81 | 0.74, 0.86 | NA | NA | NA | <0.001 |
| Total | 5 | | 452 | 0.76 | 0.65, 0.83 | 67.2% | 0.016 | REM | <0.001 |
| Late ICU (12–48 h) |  | |  |  |  |  |  |  |  |
| On-Pump | 3 | | 127 | 0.84 | 0.57, 0.95 | 89.9% | <0.001 | REM | <0.001 |
| Off-Pump | 1 | | 105 | 0.80 | 0.72, 0.86 | NA | NA | NA | <0.001 |
| Total | 4 | | 232 | 0.83 | 0.68, 0.91 | 84.9% | <0.001 | REM | <0.001 |
| Overall Periop |  | |  |  |  |  |  |  |  |
| On-Pump | 4 | | 167 | 0.76 | 0.64, 0.84 | 51.0% | 0.106 | REM | <0.001 |
| Off-Pump | 1 | | 30 | 0.46 | 0.12, 0.70 | NA | NA | NA | 0.01 |
| Total | 5 | | 197 | 0.72 | 0.57, 0.81 | 64.3% | 0.024 | REM | <0.001 |

Abbreviations: CVS, cardiovascular surgery; Early ICU, early intensive care unit period (2-8h); FEM, fixed effect model; Intraop, intraoperative period; Immed PO, immediate postoperative period; Late ICU, late intensive care unit period (12-48h); MD, mean difference; *r*, correlation coefficient; NCVS, non-cardiovascular surgery; NA, not applicable; Off-Pump, off-cardiopulmonary bypass; On-Pump, on-cardiopulmonary bypass; Overall Periop, overall perioperative period; PostInd, post-induction period; REM, random effect model; 95% CI, 95% confidence interval.
