# Supplementary material for: Correlation between central venous oxygen saturation and mixed venous oxygen saturation in surgical patients: A systematic review and meta-analysis
Source: Ann Intensive Care. 2026 May 12;16:100076. doi: 10.1016/j.aicoj.2026.100076 (PMC13195361; doi:10.1016/j.aicoj.2026.100076)
Supplement: Supplementary file 8 [file mmc8.docx]

Supplemental Table S8. Limits of agreement for ScvO₂–SvO₂: subgroup analysis

| Subgroups  Outcomes | | |  | **Surgical type** | | | | |  | | | **Circulatory support mode** | |
| --- | --- | --- | --- | --- | --- | --- | --- | --- | --- | --- | --- | --- | --- |
|  |  |  |  |  |  |  |  |  |  | | |  |  |
|  |  |  |  | CVS  NCVS | | | | |  | | | On-Pump | Off-Pump |
| LOA width and Range | | | | |  |  | |  |  |  |  | |  |
| PostInd |  | 26.22 (-14.51, 11.71) | | | | | 14.71 (-7.32, 6.39) | |  |  | 25.60 (-12.90,12.70) | | 14.00 (-8.86, 4.86) |
| Intraop |  | 25.00 (-14.44, 10.56) | | | | | 12.78 (-6.63, 8.53) | |  |  | 26.20 (-14.23,11.97) | | 15.06 (-9.81, 6.25) |
| Immed PO |  | 25.88 (-16.13, 9.75) | | | | | 14.80 (-6.45, 8.35) | |  |  | 27.86 (-15.60,12.26) | | 17.00 (-10.34, 6.66) |
| Overall Periop |  | 23.98 (-12.71, 11.27) | | | | | 15.70 (-9.10, 6.60) | |  |  | 26.48 (-14.09, 12.39) | | 13.00 (-6.99, 6.01) |

Abbreviations: CVS, cardiovascular surgery; Intraop, intraoperative period; Immed PO, immediate postoperative period; LOA, limits of agreement; MD, mean difference; NCVS, non-cardiovascular surgery; Overall Periop, overall perioperative period; PostInd, post-induction period; 95% CI, 95% confidence interval.
