# Supplementary material for: Correlation between central venous oxygen saturation and mixed venous oxygen saturation in surgical patients: A systematic review and meta-analysis
Source: Ann Intensive Care. 2026 May 12;16:100076. doi: 10.1016/j.aicoj.2026.100076 (PMC13195361; doi:10.1016/j.aicoj.2026.100076)
Supplement: Supplementary file 9 [file mmc9.docx]

Supplemental Table S9. Effect of statistical model transformation on the combined effect value of outcome indicators

| Outcomes | Heterogeneity | | | | Pooled value (95% CI) | | | Overall Effect *p* | | |
| --- | --- | --- | --- | --- | --- | --- | --- | --- | --- | --- |
|  | I^2^ | | *p* | | | FEM | REM | | FEM | REM |
| Intraop **period MD** | | 89.8% | | <0.001 | | -0.55 [-0.83, -0.28] | -1.02 [-2.20, 0.16] | | <0.001 | 0.09 |
| Intraop **period MD** | | 95.9% | | <0.001 | | 0.42 [0.13, 0.70] | -1.31 [-3.01, 0.38] | | 0.005 | 0.13 |
| Early ICU (2–8 h) **MD** | | 95.4% | | <0.001 | | 1.06 [0.34, 1.77] | -0.98 [-1.63, -0.33] | | 0.003 | 0.62 |
| Overall Periop **MD** | | 70.3% | | <0.001 | | -0.80 [-1.21, -0.38] | -0.49 [-1.34, 035] | | <0.001 | 0.25 |

Abbreviations: FEM, fixed effect model; MD, mean difference; REM, random effect model; 95% CI, 95% confidence interval.
