# Supplementary figures and images for: Correlation between central venous oxygen saturation and mixed venous oxygen saturation in surgical patients: A systematic review and meta-analysis
Source: Ann Intensive Care. 2026 May 12;16:100076. doi: 10.1016/j.aicoj.2026.100076 (PMC13195361; doi:10.1016/j.aicoj.2026.100076)

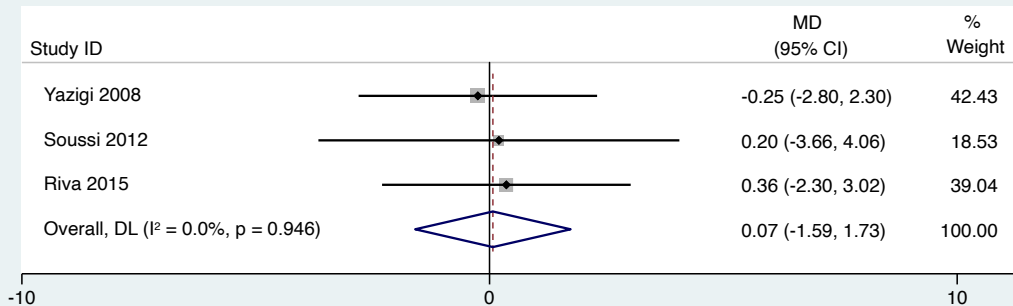

Supplement: Supplementary file 10 [file mmc10.pdf]

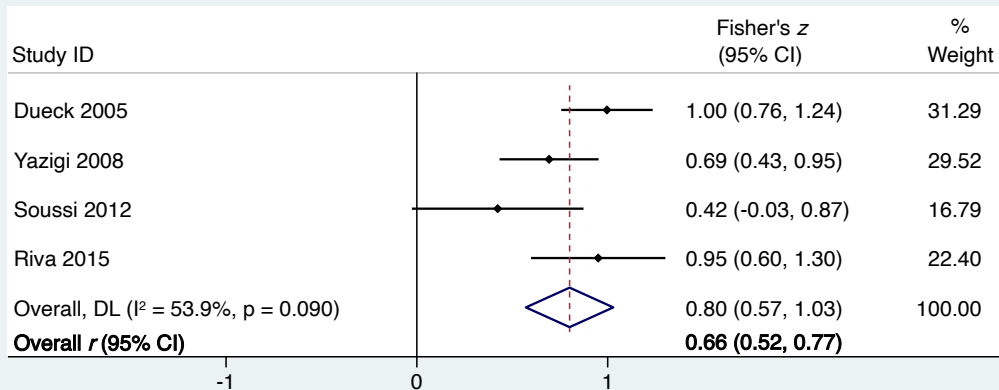

Supplement: Supplementary file 11 [file mmc11.pdf]

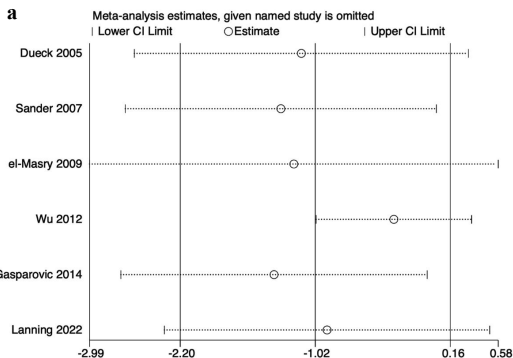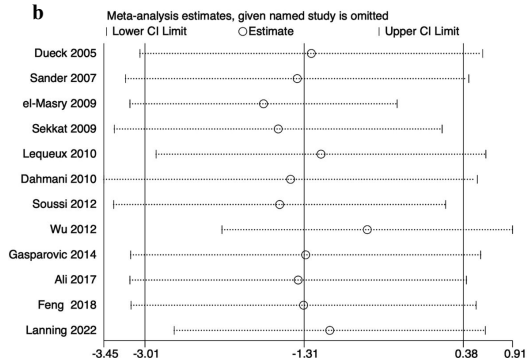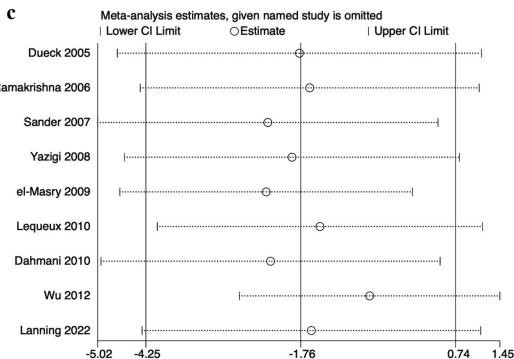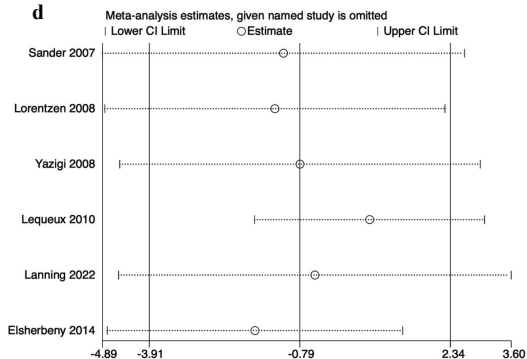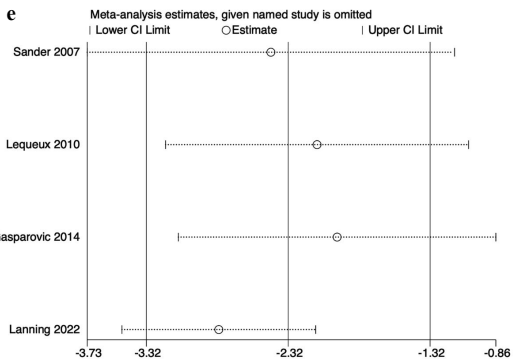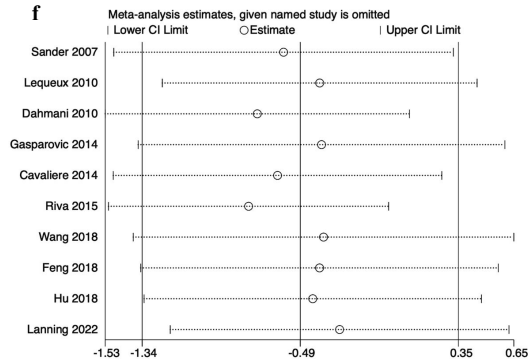

Supplement: Supplementary file 12 [file mmc12.pdf]

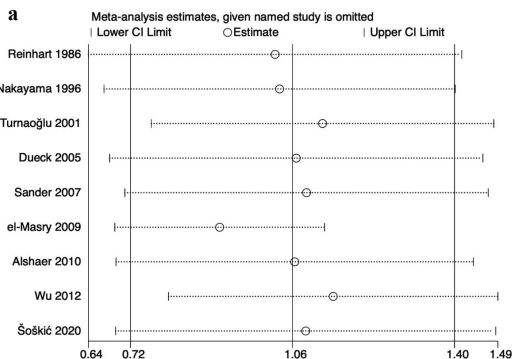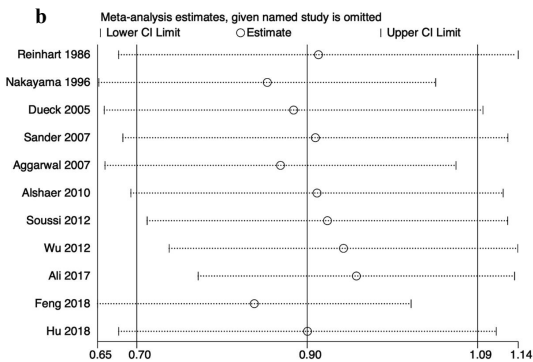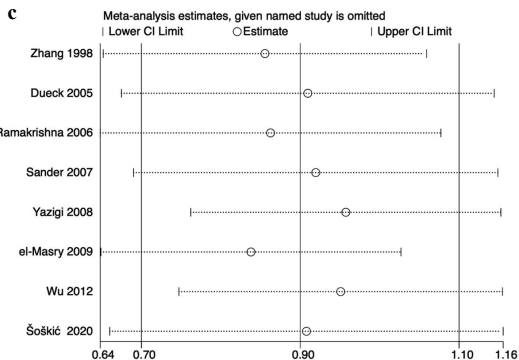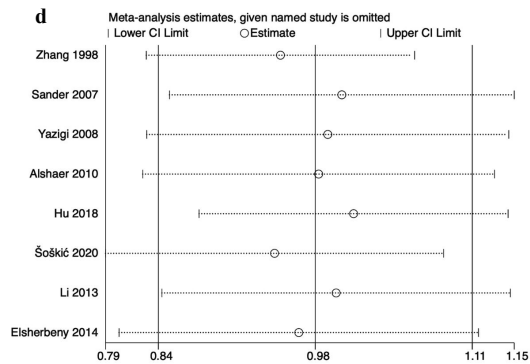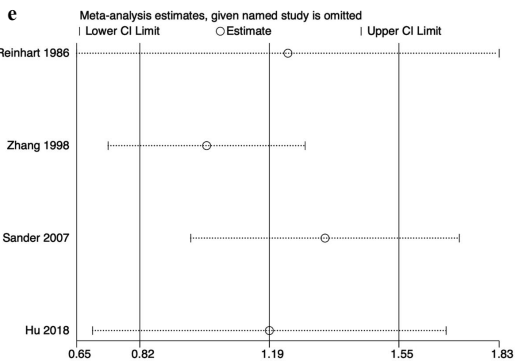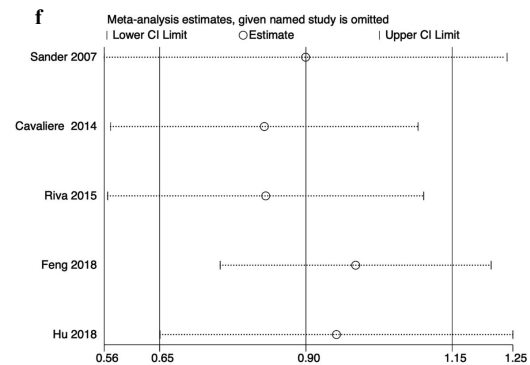

Supplement: Supplementary file 13 [file mmc13.pdf]

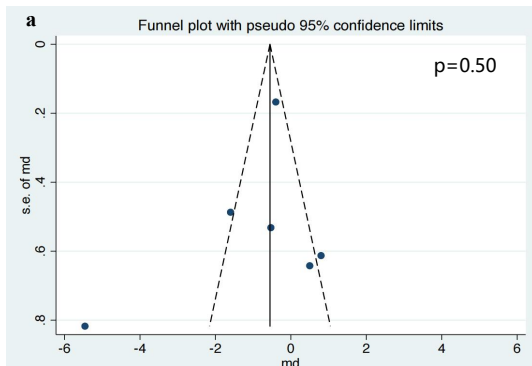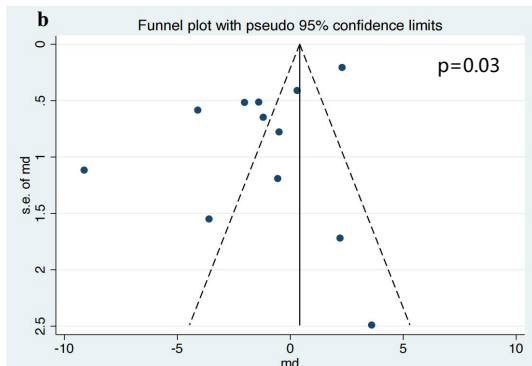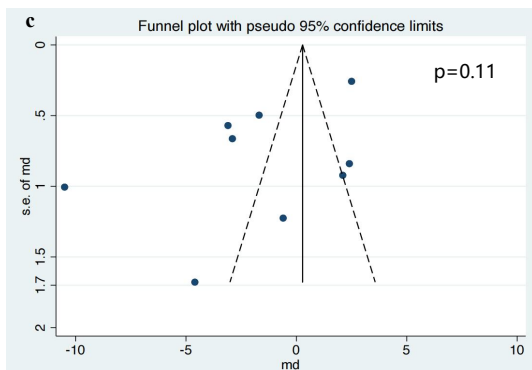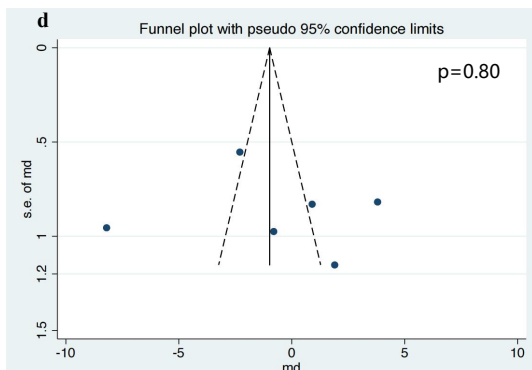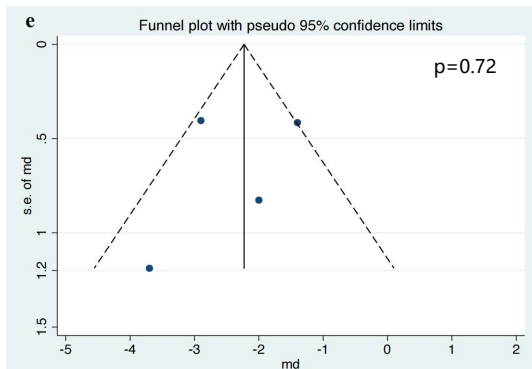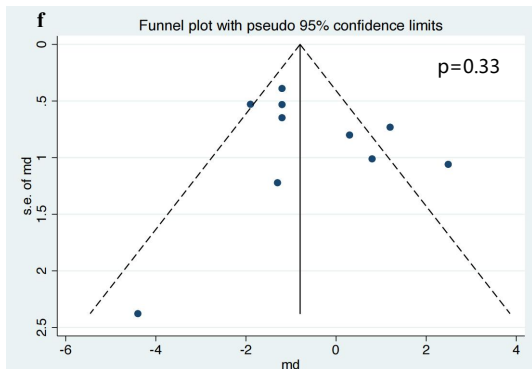

Supplement: Supplementary file 14 [file mmc14.pdf]

# Trim-and-Fill Funnel Plot

Publication Bias Assessment

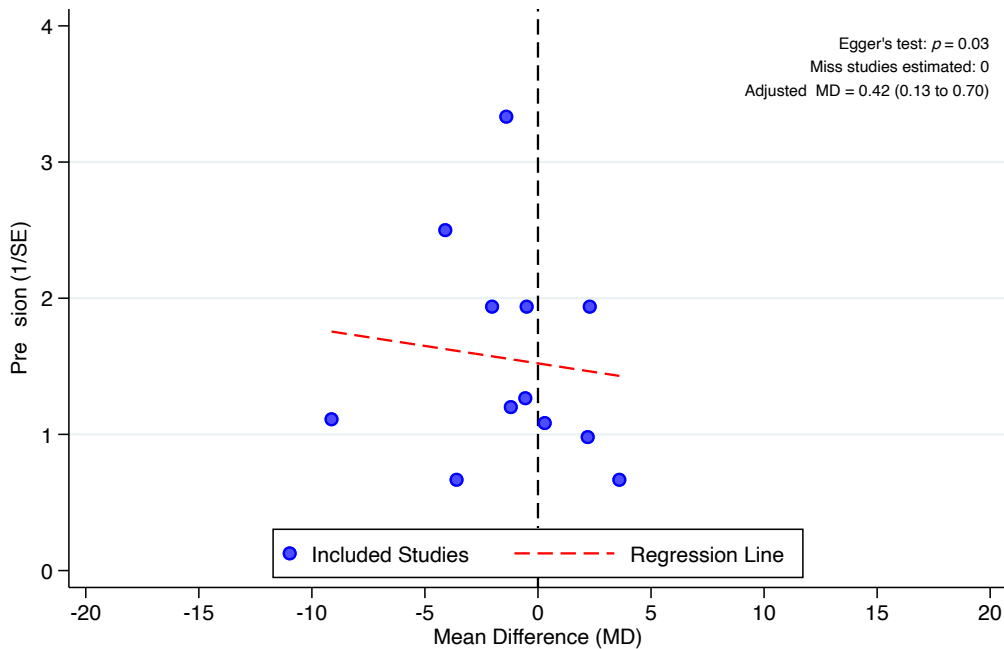

Supplement: Supplementary file 15 [file mmc15.pdf]

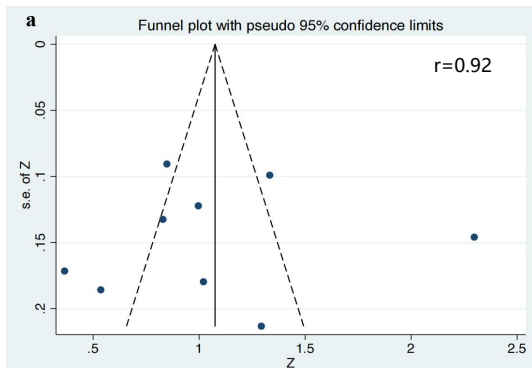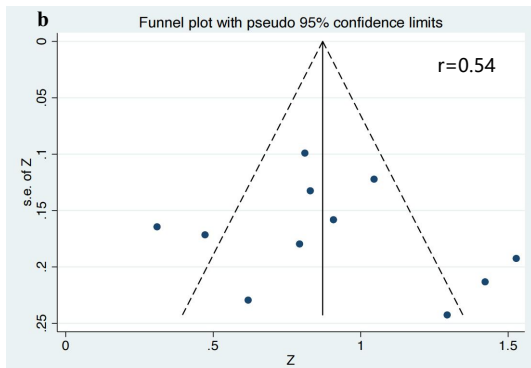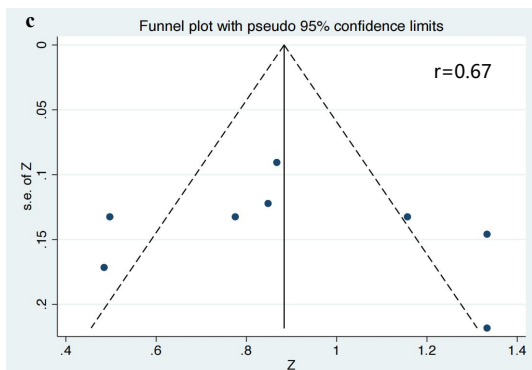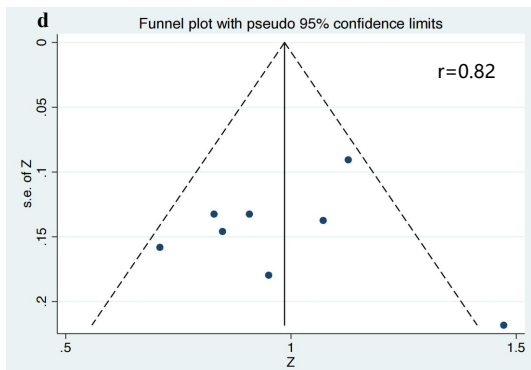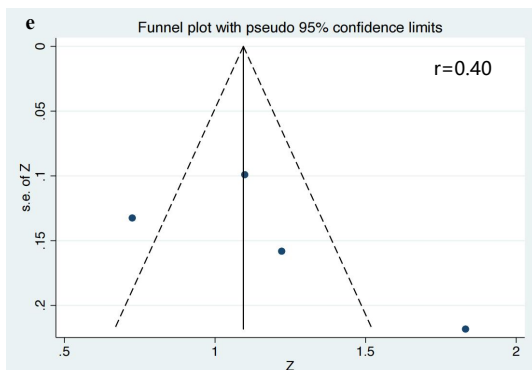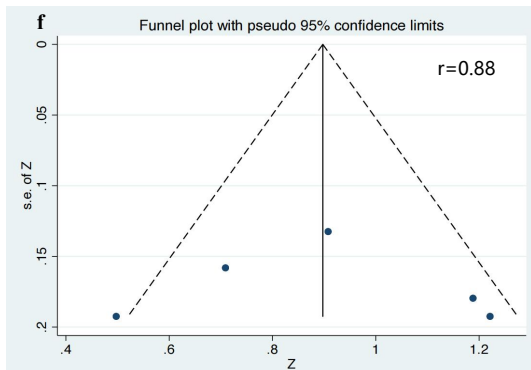

Supplement: Supplementary file 16 [file mmc16.pdf]
