# Supplementary material for: Correlation between central venous oxygen saturation and mixed venous oxygen saturation in surgical patients: A systematic review and meta-analysis
Source: Ann Intensive Care. 2026 May 12;16:100076. doi: 10.1016/j.aicoj.2026.100076 (PMC13195361; doi:10.1016/j.aicoj.2026.100076)
Supplement: Supplementary file 17 [file mmc17.docx]

**Appendix: search strategy**

| **Pubmed**  #1: **(("Oxygen Saturation"[Mesh] AND ("central venous"[TIAB] OR "scvo_2_"[TIAB] OR "scv-o_2_"[TIAB])) OR "Central Venous Oxygen Saturation"[TIAB] OR "ScvO_2_"[TIAB])**  #2: **(("Pulmonary Artery"[Mesh] AND (saturation[TIAB] OR "sv-o_2_"[TIAB] OR "svo_2_"[TIAB])) OR "Mixed Venous Oxygen Saturation"[TIAB] OR "SvO_2_"[TIAB])**  #3**:** **("Correlation of Data"[Mesh] OR correlation[TIAB] OR agreement[TIAB] OR concordance[TIAB] OR "Bland-Altman"[TIAB])**  #4: #1 AND #2 AND #3 | 53 |
| --- | --- |
| **Cochrane Library**  #1: (ScvO_2_ OR Scvo_2_ OR "central venous oxygen saturation")  #2: (SvO_2_ OR Svo_2_ OR "mixed venous oxygen saturation")  #3: (comparison OR correlation OR agreement OR concordance OR "Bland-Altman")  **#4:** #1 AND #2 AND #3 | 6 |
| **OVID**  #1: (exp central venous oxygen saturation/ or ("central venous oxygen saturation" or ScvO2 or Scvo2).ti,ab,kw.  #2: exp mixed venous oxygen saturation/ or ("mixed venous oxygen saturation" or SvO2 or Svo2 or "pulmonary artery oxygen saturation").ti,ab,kw.  #3: (correlat* or compar* or agreement or "Bland-Altman" or substitut* or equivalen* or concordance).ti,ab,kw.  #4: exp surgical patient/ or exp perioperative period/ or exp surgical procedure/ or (surg* or operat* or perioperat* or postoperat* or intraoperat* or "post-operative" or "intra-operative").ti,ab,kw.  #5: #1 AND #2 AND #3 AND #4 | 47 |
| **Embase**  ('central venous oxygen saturation'/exp OR 'central venous oxygen saturation' OR 'scvo_2_':ti,ab,kw OR 'central venous o_2_ saturation':ti,ab,kw) AND ('mixed venous oxygen saturation'/exp OR 'mixed venous oxygen saturation' OR 'svo_2_':ti,ab,kw OR 'mixed venous o_2_ saturation':ti,ab,kw) AND ('comparative study'/exp OR 'comparative study' OR 'method comparison':ti,ab,kw OR 'bland-altman':ti,ab,kw OR 'limits of agreement':ti,ab,kw OR 'concordance':ti,ab,kw OR 'coefficient of correlation':ti,ab,kw) | 38 |
| **CNKI**  (SU=('Central Venous Oxygen Saturation' + 'ScvO2')) AND (SU=('Mixed Venous Oxygen Saturation' + 'SvO2')) | 42 |
| **Wanfang data**  ("central venous oxygen saturation" OR "ScvO2" OR "central venous oxygen saturations") AND ("mixed venous oxygen saturation" OR "SvO2" OR "mixed venous oxygen saturations") AND ("correlation" OR "relationship" OR "comparison" OR "agreement" OR "Bland-Altman") NOT ("Case Report" OR "Review" OR "Meta-Analysis") | 23 |
| **Weipu**  (TI=("central venous oxygen saturation" OR "ScvO2") AND TI=("mixed venous oxygen saturation" OR "SvO2")) OR (KY=("central venous oxygen saturation" OR "ScvO2") AND KY=("mixed venous oxygen saturation" OR "SvO2") | 25 |
| **SinoMed**  ("central venous oxygen saturation" OR "central venous oxygen saturations" OR ScvO_2_) AND ("mixed venous oxygen saturation" OR "mixed venous oxygen saturations" OR SvO_2_) AND (correlation OR correlations OR relationship OR agreement OR concordance OR comparison) | 13 |
| **Total** | 247 |
